# Behavioral Deficits in Animal Models of Blast Traumatic Brain Injury

**DOI:** 10.3389/fneur.2020.00990

**Published:** 2020-09-04

**Authors:** Aswati Aravind, Arun Reddy Ravula, Namas Chandra, Bryan J. Pfister

**Affiliations:** Department of Biomedical Engineering, Center for Injury Biomechanics, Materials and Medicine, New Jersey Institute of Technology, Newark, NJ, United States

**Keywords:** blast TBI, behavior deficits, cognitive deficits, anxiety and depression, motor deficits, auditory deficits, fear conditioning

## Abstract

Blast exposure has been identified to be the most common cause for traumatic brain injury (TBI) in soldiers. Over the years, rodent models to mimic blast exposures and the behavioral outcomes observed in veterans have been developed extensively. However, blast tube design and varying experimental parameters lead to inconsistencies in the behavioral outcomes reported across research laboratories. This review aims to curate the behavioral outcomes reported in rodent models of blast TBI using shockwave tubes or open field detonations between the years 2008–2019 and highlight the important experimental parameters that affect behavioral outcome. Further, we discuss the role of various design parameters of the blast tube that can affect the nature of blast exposure experienced by the rodents. Finally, we assess the most common behavioral tests done to measure cognitive, motor, anxiety, auditory, and fear conditioning deficits in blast TBI (bTBI) and discuss the advantages and disadvantages of these tests.

## Introduction

Traumatic Brain Injury (TBI) is one of the most prevalent causes for disability and a reduced quality of life among military personnel. About 380,000 military personnel have been diagnosed with TBI since 2000 according to the Defense and Veterans Brain Injury Center (DVBIC) (1). Of these, more than 50% of combat TBI are reported to occur due to blast exposures (2). Most of the soldiers with blast TBI (bTBI) develop cognitive, behavioral and psychological deficits such as PTSD, attention deficits, headaches, anxiety, depression, memory loss, suicidal ideation, tinnitus and vestibular deficiencies (3–6). The etiology of these deficits following a blast injury is unclear. One of the major challenges over the years of bTBI research has been to design a blast injury model in rodents that mimics the primary blast wave and correlates to the clinical outcome of human TBI.

Blast loading is typically classified into four categories: (1) Primary effect caused by the blast wave propagating through the skull and brain, (2) Secondary effects caused by the penetration of shrapnel, (3) Tertiary effects caused by the blast wind resulting in impact or acceleration-deceleration injuries and (4) Quaternary effects caused by toxic gases from the blast exposure (7). The primary effect of a blast exposure caused by the propagation of the blast wave through the brain has been the main area of focus in the area of blast induced neurotrauma. Linking blast exposure mechanics to the behavioral outcome is crucial in validating rodent blast models in order to the study the underlying cellular mechanisms and to further drug development, as exposure level determines injury severity and hence the mechanisms. Shock tubes have been designed to induce bTBI in rodent models since 2007 (8). Several variants of shock tube models have been used in the past 10 years to study behavioral outcome of bTBI in rodent models. A recent review from our group has listed the types of shock tubes used in bTBI with some of the geometric, process and test parameters (9). The variations in those parameters result in inconsistent findings of behavioral outcome (9).

The objective of this review is (1) to outline and evaluate the behavioral tests currently being used in bTBI (2) to summarize the parameters used in testing and the deficits observed in rodents subjected to blast injury between 2008 and 2019. (3) to highlight the crucial features in the blast testing that can lead to the variability in behavioral deficits seen throughout the bTBI literature.

## Overview of Blast Apparatus in rodent Models

The major criterion for a blast tube is to reproduce the biomechanical loading of an open field blast exposure and mimic the clinical outcome observed in TBI patients. A blast overpressure wave propagates as a sharp positive pressure rise compressing the surrounding medium of air or water moving radially outward followed by a negative under pressure wave before returning to baseline or ambient pressure values. The primary blast pressure wave generated in open field explosions has been best described by the Friedlander waveform (10). Pressure waves of explosions in confined spaces are more complex due to reflection of the wave from objects, ground, diffraction, and interaction with the incident shockwave. Most shock tubes used to study rodent models of bTBI have attempted to recreate the Friedlander waveform in order to allow for better reproducibility and comparison across laboratories (10, 11).

One of the most direct forms of bTBI exposure in rodent models has been subjecting the animals to open field blast explosions using TNT or other explosives. This model helps to mimic real life blast exposures containing the primary, reflected waveforms and blast wind. The open field explosions expose the rodents to primary, secondary, tertiary, and quaternary effects of the blast. As a result, these models are associated with higher mortality and difficulty in controlling the clinical outcome in the animals. Open field experiments also require large number of explosives to generate the desired blast pressures when compared to recreating the same blast pressures in shock tubes (12–14). In the 1950s, Clemedson and Criborn (15), proposed a cone shaped shock tube using pentaerythritol tetranitrate (PETN) explosives as a blast injury model for rats. The rats were placed 1 m away from the source of detonation and were fixed with the help of metallic nets to limit the tertiary effects of the blast. This, however, does not limit the effects of reflective wave on the rodent head. This model also did not negate the quaternary effects of the blast but required less explosives than the open field experiments to produce the desired blast overpressure (8, 11, 13, 15).

**Figure 1:**
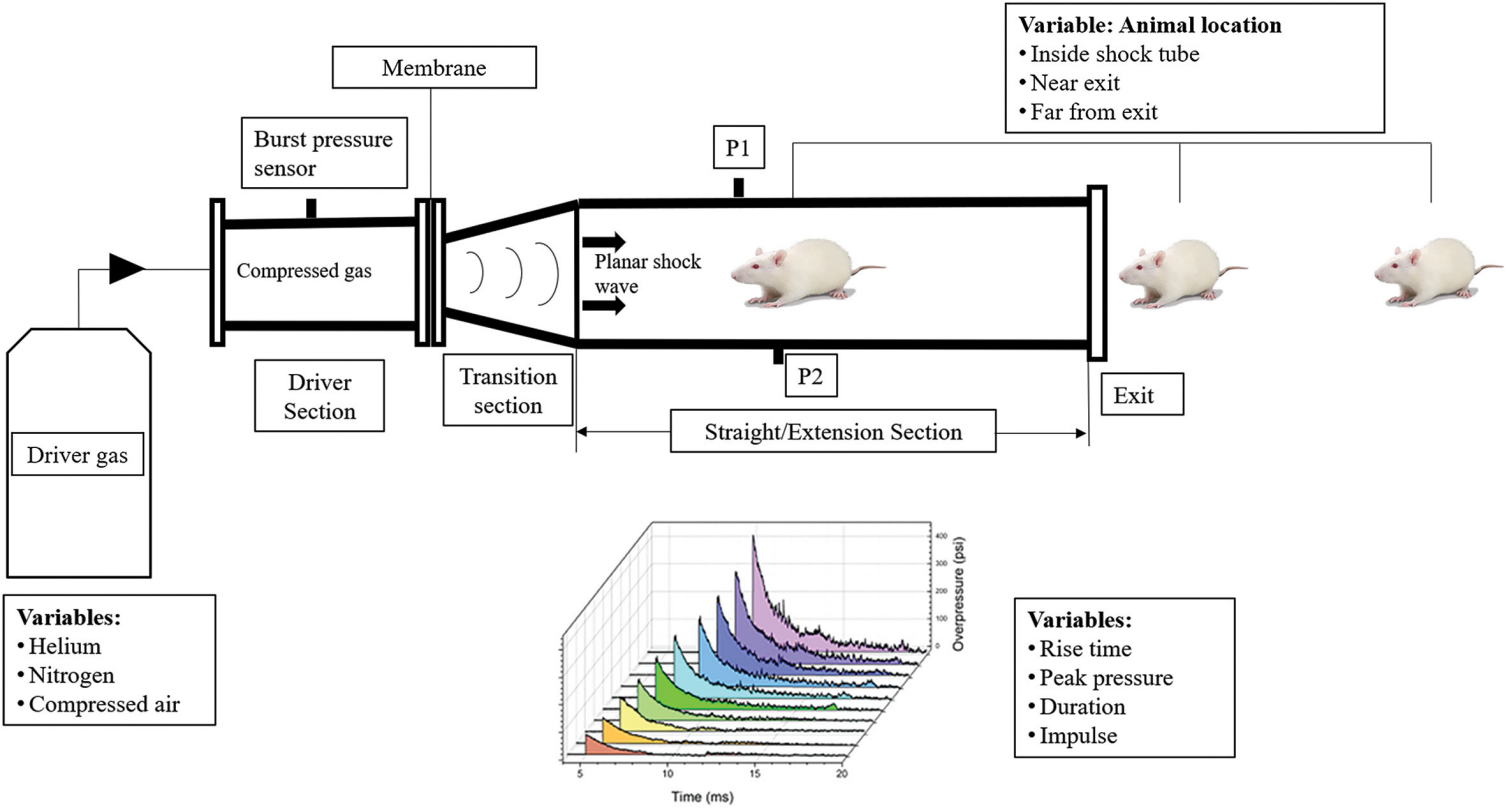
Variability in the design and process variables of the shock tube and its effect on shock wave profile, bTBI injury and behavior in animal models. The compressed gas and number of membranes used affect the incident pressure generated in shock tubes. The geometry of the shock tube, presence or absence of a transition section affect variables of the shockwave profile such as rise time, peak pressure, duration, and impulse. The animal locations inside, near exit and far from exit of the blast tube play a role in the injury severity and behavioral outcomes. P1 and P2 represent the side on blast pressure measurement sensors.

In order to study the effects of the primary blast wave, shockwave tubes using compressed gases that disrupt membranes in order to produce a planar Friedlander waveform have been used (10, 16). Such shock tubes typically contain a driver section that is filled with a compressed gas usually helium, nitrogen or compressed air separated by membranes from the transition and extension section, where the animal is placed, followed by an end plate as shown in Figure 1. The compressed gases fill the driver section leading to the disruption of the membranes. The rapidly expanding gas propagates as a planar shock wave front down the test section. The number, thickness of the membranes and length of the test section controls the intensity, duration, and impulse of the blast overpressure wave produced. Several variations of these models are currently in use for investigating bTBI in rodents (10, 17–19).

Some shock tube variations that can alter behavioral outcomes include position of the animal in the blast tube, orientation of the animal, use of head restraint, end conditions of the blast tube (open vs. closed) and length of the blast tube (20). The animal's position in the blast tube plays a crucial role in determining the type of blast injury it is exposed to. Several studies have shown that animal placement at the exit of the blast tube leads to significant changes of the incident pressure waveform from the ideal Friedlander waveform (10, 21). At the outer edges of the blast tube near the exit end, an expansion wave is created that decreases the peak pressure and impulse of the blast overpressure wave, exposing the rat brain to a subsonic jet wind resulting in an acceleration-deceleration induced tertiary blast injury. The animals that were constrained and placed at the exit end of the blast tube experienced high amounts of compression pressure in the head and neck regions (10, 21–24).

Another criterion that is crucial in the design of the blast tube is the closed or open exit end of shock tubes. A closed ended shock tube results in the propagation of a reflected wave back into the shock tube when the incident pressure wave reaches the end of the tube. This results in the rodent being exposed to a complex blast waveform with the gases unable to dissipate from the blast tube. An open-ended shock tube, on the other hand, results in the formation of an expansion wave at the end of the shock tube which propagates back into the tube (25). Kuriakose et al., indicated that an end plate reflector placed at an optimum distance to the exit of the blast tube can reduce the reflective waveforms and thereby prevent secondary loading (7). Absence of head restraint during the blast exposure also results in tertiary effects of blast leading to increased behavioral deficits (18, 26). The blast overpressure waves decays over the length of the blast tube. The optimal placement of the animal in the blast tube would be where the Friedlander wave is fully formed (10, 27). The amplitude of the incident blast overpressure wave is also critical in determining the severity in terms of mild, moderate or severe TBI. Previous work in our lab has classified the severity of bTBI overpressure wave based on animal mortality and presence of oxidative markers (28). Due to different blast models used across labs, varying lengths of blast tube and thereby incident blast overpressures corresponding to mild, moderate, and severe bTBI have been reported in literature (28–31).

## Behavioral Deficits in Blast TBI

### Cognitive Deficits

Cognitive deficits have been reported in patients with mild TBI which often resolve in 6 months while moderate and severe TBI cause long term deficits including dementia or other neurodegenerative disorders (32). Soldiers exposed to bTBI have been diagnosed with retrograde or anterograde amnesia and decreased executive function (33, 34). Cognitive deficits, however, have been more associated with blunt TBI than with a primary blast exposure (32). In a study by Barnes et al., it was reported that older veterans diagnosed with a TBI were associated with a 60% increase in the risk of developing dementia over a period of 9 years (35).

bTBI in animal models have been reported to affect prefrontal and hippocampal regions involved in cognition (36). Recognition memory in rodents following bTBI has been evaluated using the novel object recognition test while spatial working memory has been studied using Morris water maze (MWM), Barnes maze and Y maze tests. Novel object recognition test (NOR) measures deficits in both the perirhinal cortex which plays a major role in object recognition and the parahippocampal region involved with visual object recognition memory. NOR measures the ability of the rodent to recognize novelty and is measured by the difference in time spent by the animal in exploring a novel object when compared to a familiar object. The main advantage of the NOR test is its simplicity and lack of training or habituation required prior to conducting the test. The NOR test can also be modified to study short or long term memory deficits by increasing or decreasing the retention time before introducing a novel object (37, 38).

Allocentric spatial memory involving the hippocampus and entorhinal cortex has been studied using several maze tests with the most popular form of assessment being the Morris Water Maze test (39). The other alternatives heavily used in the blast literature have been the Barnes Maze, radial arm maze and Y maze tests. Morris water maze tests the ability of a rodent to use spatial visual cues to identify the location of a rescue platform submerged in water. Place cells present in the hippocampus and entorhinal cortex have been identified to have increased firing rates when the rodent nears the submerged platform location in the MWM test (40). Further, lesions in the hippocampus have been identified to eliminate water maze learning (41).

The Barnes Maze test is similar to the MWM in identifying hippocampal related deficits in cognition. Barnes maze test consists of a circular platform with evenly spaced holes around the circumference with an escape platform placed under one of these holes. The presence of brightly lit open space, use of an aversive sound or blowing air above the maze are some of the methods used to motivate the rats to seek the escape platform (20, 39). The advantage of the Barnes maze test over MWM is that it does not induce the stress of swimming in the animals as measured by the increased plasma cortisone levels in the rats post MWM test (42).

The Y maze test is also used to measure deficits in the hippocampus and the prefrontal cortex and can be used to measure the spatial working and reference memory. The Y maze consists of 3 arms, 120° to each other. Similar to the RA, the number of entries to each arm is measured with greater alternation in the arms indicating good working memory. An alternative to this approach is closing one of the arms during training and measuring the spatial reference memory of the rats to visit the novel unvisited arm during the testing phase. Recognizing the previously visited arms with spatial cues and increased visits to the novel arm indicates intact hippocampal function (43).

Findings of cognitive deficits recorded by researchers across different models of shockwave tubes and blast pressures are summarized in Table 1. Literature review was done using advanced search in Google Scholar for articles with all of the words “rodents, shockwave tube, cognitive memory deficit” and the exact phrase “blast TBI,” between the years 2008–2019. Studies generating blast TBI conditions using open field detonations or shockwave tubes were only incorporated in this review.

**Table 1:** Summary of experimental variable and cognitive deficits observed in rodent models of blast TBI.

**Behavior test**	**Animals**	**Position of animal**	**Head restrained**	**Gas used in blast tube**	**Blast pressure**	**Duration of deficits**	**References**
Morris Water Maze (MWM)	Wistar Rats (230–330 g)	0.25 m from expansion compartment facing the blast wind	Yes	Compressed air.	10 and 30 kPa at 4–6 ms	Up to day 2 for both 10 and 30 kPa exposure groups	(44, 45)
	Sprague-Dawley (SD) rats	39 and 17 cm outside shock tube opening, 40° and 20° lateral to shock tube axis respectively	Yes	Helium	100 and 450 kPa reflected peak overpressure	Up to 30 days for both groups	(46)
	SD rats (250–350 g)	Transverse prone position across the mouth of the blast tube.	Yes. Chest protection with rat head exposed	Compressed air	114, 126, and 147 kPa	Days 8 and 9	(30)
	SD rats (225–250 g)	Supine position with the blast wave generator nozzle above the animal's head.	Yes, chest shielded with 2 mm lead plate	Nitrogen	80 Psi	Up to day 13–15 post blast.	(31)
	Male SD rats (150–200 g)	265 mm from the exploding wire with head facing impact	No; movement restricted	Exploding wire technique to generate small scale blast waves	95 kPa for 0.189 ms	No deficits from 8 to 11 days post blast. In the reversal phase test deficits were observed on days 13–15	(47)
	Male SD rats (250 g)	1.09 m inside the shock tube facing impact	No information	Compressed helium	97, 117, 153 kPa	Deficits for 117 and 153 kPa were observed at 48 h but not at 3, 6, or 72 h post blast	(17)
	SD rats (289 ± 21 g)	17 cm from the opening of the tube at an angle of 18° to shock tube axis with the sagittal plane of rat head perpendicular to the radial axis of shock tube.	Yes	Helium	450 kPa for 0.4 ms	No deficits observed up to 60 days post blast	(48)
	Long Evans hooded (LE) rats (250–350 g; 10–12 weeks of age)	18 inches inside the shock tube. Animals were placed both facing and sideways to the blast.	Yes	Compressed air	36.45 ± 2.32 kPa, with a mean duration of 3.78 ± 0.09 ms. One blast every day for 12 days over 2.5 weeks	Significant deficits 24 h. post the last blast impact	(49)
	LE rats (250–350 g, 10 weeks old)	30 cm inside the shock tube with head facing the blast exposure	Yes	Compressed air	74.5 kPa for 3 days	No memory deficits at 9 weeks post blast	(50)
	LE rats (250–300 g)	18 inches inside the shock tube. Animals were placed both frontal and sideways to the BOP	Yes	Compressed air	Repeated 36.6 kPa for 12 days	24 h after blast animals exposed at a frontal orientation showed slower acquisition memory at the 2nd and 3rd trial of the MWM test.	(18)
	SD rats (250–300 g)	4 cm from the blast cone	Minimal spatial movement of head	Compressed Nitrogen	80 Psi	Days 5 and 6 post blast	(51)
	C57BL/6 mice (2–3 months, 20–24 g)	Not mentioned	Not mentioned	Compressed air	321.2 ± 4.01 kPa at 50.29 ± 1.86	Up to 8 weeks post blast	(52)
	SD rats (60–70 days)	503 cm from the bursting membrane and 112 cm from the open end of the driven cylinder	Yes, with no earplugs. Head restraint not mentioned	Helium	14 Psi	No deficits 5 weeks post blast.	(53)
	SD rats (6-month-old)	Placed outside with right side of the skull facing the pressure wave	No	Nitrogen	50 Psi in 1 ms	At 3 weeks post blast	(54)
Barnes maze	Heterozygous *WldS* mice (8-week-old)	10 cm from Mylar membrane, with the left side of head facing the blast	No	Compressed air	27 kPa	On day 11 post blast	(55)
	C57BL/6 Mice (2.5 months)	0.56 m inside open exit of shock tube	Both with and without head restraint	Compressed gas	77 ± 2 kPag	Up to 5 days (Deficits were resolved when mice heads were restrained during blast)	(26)
	SD Rats (~300g)	Transverse prone position across the mouth of the blast tube.	Yes, with chest protection	Compressed air	20.6 ± 3 psi	Days 10 and 11 in test session 1 (conducted between 10 and 15 days post injury) and all days of test session II (between 47 and 51 days post injury)	(56)
	SD rats (300–330 g)	Transverse prone position across the mouth of the blast tube.	Yes. Chest protection with rat head exposed	Compressed air	138 kPa single or 5 blasts	No significant deficits up to day 21 post blast	(19)
	SD Rats (~300 g)	Transverse prone position across the mouth of the blast tube.	Yes, with chest protection and head exposed	Compressed air	20.63 psi. Animals were placed in normal or environmentally enriched housing post blast	Animals in normal housing had deficits on day 16, 18 and days 67–71 post impact. Blast animals in enriched housing had no deficits.	(57)
	C57BL/6J mice (2-month-old)	Prone position 3-m distance away from the source with the head longitudinally aligned to the shockwave propagation	No	C4 explosives	~46.6 kPa	23–29 days post injury in the 3 m blast group	(58)
Eight arm radial maze	LE rats(250–350 g, 10 weeks old)	30 cm inside the shock tube with head facing the blast exposure	Yes	Compressed air	74.5 kPa for 3 days	No deficits at 10–23 weeks post blast	(50)
Y Maze	ICR Mice (30–40 g)	7 m from open detonation source	No	500 g TNT	17.2 kPa Shockwave and reflected wave	No deficits	(59)
	ICR Mice (25–30 g)	4 and 7 m away from detonation source	No	500 g TNT	Shockwave and reflected wave 2.5Psi or 5.5 Psi	At 7 day in the 2.5 Psi group. 7 and 30 days in the 5.5 Psi group animals.	(12)
Novel Object Recognition (NOR)	ICR mice (25–30 g)	4 and 7 m away from detonation source	No	500 g TNT	Shockwave and reflected wave 2.5 or 5.5 Psi	7 and 30 days after blast exposure for 2.5 and 5.5 Psi blasts.	(12)
	ICR Mice (30–40 g)	7 m from open detonation source	No	500g TNT	17.2 kPa Shockwave and reflected wave	Up to 14 days	(59)
	ICR mice (30–40 g)	7m from detonation source, placed ‘side on’ to the blast source.	No	500g of TNT	17.23 kPa	Deficits observed on days 7 and 14 post blast	(60)
	SD Rats (250 g)	Rostral cephalic orientation facing the shockwave inside the tube	Not mentioned	Compressed helium	129.23 ± 3.01 kPa for 2.5 ms	2 weeks	(61)
	LE rats (250–350 g, 10–12 weeks old)	Head facing the shockwave exposure	Yes	Compressed air	One 74.5 kPa exposure for 3 consecutive days	Between weeks 11–17 post blast	(62)
	SD rats (200–250 g)	Prone position 20 cm from the end of the tube facing the blast wave	No	Compressed air	74 kPa	2 weeks post blast impact.	(63)
	SD rats (250 g)	1.09 m from open end of the blast tube in a rostral cephalic position	No information on head restraint	Helium	117 kPa at 2.5 ms	Significant deficits at 1- and 3-months post impact	(36)
	SD rats (250 g)	1.09 m from open end of the blast tube in a rostral cephalic position	No information on head restraint	Helium	117 kPa at 2.5 ms	At 7 days post blast but not at 3 days.	(64)
	LE rats (250 g−350 g; 10 weeks of age)	Head facing the blast exposure	Yes	Compressed air	74.5 kPa	At 33 weeks post blast	(65)
Spatial Object Recognition (SOR)	Adult C57BL/6 mice (12–16 weeks)	1 cm outside exit end of blast tube facing head front.	Both head restrained and unrestrained	Helium	Mild blast of 215 ± 13 kPa, 46 ± 5kPa * ms impulse or moderate blast of 415 ± 41 kPa, 148 ± 12 kPa * ms impulse	Both blast groups showed deficits 5 days post blast. No difference was observed between mild vs. moderate blast or head restrained vs. unrestrained group.	(66)
Active avoidance test	Male C57/Bl6 mice (3–4 months; 25.22 ± 1.96 g)	Supine position 53 cm from driven section opening	Yes	Helium	Rupture pressure of 183 kPa(mild) or 213 kPa (moderate)	Mild blast animals showed deficits up to day 21 post blast but not on day 30. Moderate blast had deficits till day 30.	(16)
	Wistar rats (220–250 g)	Not mentioned	Not mentioned	Compressed air	338.9 ± 9.1 kPa at 52 ms	Up to 5 days	(29)
Passive avoidance task	LE rats (250–300 g)	18 inches inside the shock tube, in both frontal and sideways orientation to the blast	Yes	Compressed air	36.6, 74.5, and 116.7 kPa	Up to 24 hrs. in the 116.7 kPa in sideways orientation and 74.5 kPa frontal orientation when animals were trained after blast exposure	(18)
	ICR Mice (30–40 g)	7 m from open detonation source	No	500 g TNT	17.2 kPa Shockwave and reflected wave	No deficits	(59)
Visual- cue discrimination	SD rats	17 cm from the end of the shock tube	Yes	Helium	450 kPa over 80 kPa*ms	24 h post injury	(67)

Based on observations from Table 1, deficits in object recognition memory has been consistently reported in bTBI using the novel object recognition test. Deficits in recognition memory are reported in mild blast exposures of 74–129 kPa and last from 7 days up to 33 weeks following bTBI. Interestingly, Sajja et al. (64), did not observe novel object recognition deficits on day 3 following blast but the animals were observed to develop the deficits on day 7 post injury. This could indicate that the recognition memory deficit in bTBI is not immediate but occurs over time.

Spatial working memory deficits have not been consistently reported across bTBI models. Some factors that contribute to the difference in outcome are the positioning of the animal in the blast tube, blast pressures exposed, use of head restraint and the overall design of the blast tube. Working memory deficits measured by Y maze test has been reported only in animals subjected to open detonations and the duration of deficits directly correlate with the distance from the detonation source (12). Similarly, Barnes maze test has only been reported in literature for injured animals without head restraint or animals kept outside the exit end of the blast tube. These deficits were observed in low bTBI pressures and lasted up to 11 days post blast. Memory deficits in open detonation experiments persisted for a month (58). The working memory deficits in Barnes maze were resolved when animals were provided with head restraint (26). Most deficits that lasted weeks post injury observed in the Morris Water maze test also involves animals placed at the exit end of the blast tube. When the animals are placed inside the blast tube only transient deficits observed at 24 or 48 h post injury were observed (17, 18).

### Anxiety and Depression

Veterans diagnosed with bTBI also report developing anxiety and depression disorders over time. In a sample population of veterans exposed to bTBI, it was identified that 50% of individuals exposed to bTBI developed anxiety disorders, 88% developed depression and 60% showed Post Traumatic Stress Disorder (PTSD) traits (68). Treating military personnel with PTSD and depression has been estimated to cost ~$6.2 billion in the first 2 years post deployment (69).

Depression is a complex mental illness which causes persistent feeling of sadness and loss of interest which affects person's quality of life negatively. Severe post-concussive symptoms such as headache, blurred vision, dizziness, and memory impairment manifests due to depression following TBI. Depression following TBI causes disturbances in neuronal circuits such as frontal lobe -basal ganglia circuits and anterior ascending monoaminergic pathways (70, 71). The forced swim test is one of the popular behavior tasks successfully used for assessing depression behavior. Mice or rats are placed in an inescapable transparent tank that is filled with water and their escape related mobility behavior is measured in order to evaluate depression. Increased time spent immobile in the tank correlates with depression traits such as behavioral despair and disengagement from stress coping (72). Can et al. (72), Slattery et al. (73), and Yankelevitch-Yahav et al. (74) have provided detailed protocols for the test parameters for forced swim test for rats and mice (72–74).

Anxiety is a psychological, physiological and behavioral state induced in animal or humans by a threat to well-being. Anxiety is characterized by an increased arousal, expectancy, autonomic and neuroendocrinal activation which results in manifestation of specific pattern of behaviors such as exploration, feeding, flight or defensive behavior to the specific stimulus (75). Anxiety behavior is regulated by forebrain and hindbrain regions involving the septo-hippocampal system, entorhinal cortex, medial prefrontal cortex, basolateral amygdaloid complex, and midbrain raphe system (76–80).

Anxiety in rodent models of bTBI has been evaluated using tests such as the elevated plus maze (EPM), open field test, elevated zero maze and light/dark box tests as described in Table 2. The EPM test is widely used to study anxiety and has been used to identify deficits in regions such as the limbic regions, hippocampus, amygdala and dorsal raphe nucleus (31, 47, 48, 56, 83, 90). Briefly, rats or mice are placed at the junction of the four arms of the maze, two of which are closed by walls and two are open. The animal is placed facing an open arm and the time spent or number of entries in the open or closed arms of the maze is recorded. The time spent in the closed arms correlates to anxiety, since the animal lacks the drive to explore the open arm that is less safe. The elevated zero maze works on a similar principle as the elevated plus maze with closed and open arms. The elevated zero maze is circular with alternating open and closed arms.

**Table 2:** Summary of experimental variables and anxiety and depression related behavior test outcomes in bTBI.

**Behavior test**	**Rodent used**	**Position of animal**	**Animal restrained**	**Gas used in blast tube**	**Blast pressure**	**Duration of deficits**	**References**
Elevated Plus Maze (EPM)	ICR mice (30–40 g)	7 m away from detonation source	No	500 g TNT	17.2 kPa Shockwave and reflected wave	No deficits observed at day 7	(59)
	SD rats (~300 g)	Transverse prone position across the mouth of the blast tube.	Yes, with chest protection	Compressed air	20.6 ± 3 psi	No deficits at 9 days but deficits were observed 46 days post blast.	(56)
	SD rats	39 and 17 cm outside shock tube opening, 40 and 20° lateral to shock tube axis, respectively	Yes	Helium	100 and 450 kPa reflected peak overpressure	No deficits at 4 days or 30 days post impact	(46)
	Male SD rats (150–200 g)	265 mm from the exploding wire with head facing impact	No	Exploding wire technique to generate small scale blast waves	95 kPa for 0.189 ms	At days 38 and 62 post blast	(47)
	SD rats (225–250 g)	Supine position with the blast wave generator nozzle right above the animal's head.	Yes, chest shielded with 2 mm lead plate	Nitrogen	80 Psi	Deficits at day 9 but no deficits at days 22 and 48 post blast.	(31)
	Male Wistar rats (408.3 ± 93 g)	Right side of the front cortex is aligned to the impact	Yes	A lithotripsy machine fired 5 shockwave pulses with 60 Hz frequency	~50 MPa	Significant deficits post blast impact	(81)
	SD rats (289 ± 21 g)	17 cm from the exit of the tube at an angle of 18° to shock tube axis with the sagittal plane of rat head perpendicular to the radial axis of shock tube.	Yes	Helium	450 kPa for 0.4 ms	At 60 days but not at 30 days	(48)
	ICR mice (30–40 g)	7 m from detonation source. Animals were placed ‘side on’ to the blast source.	No	500g of TNT	17.23 kPa	No deficits were observed on days 7 and 14 post blast	(60)
	SD rats (~300 g)	Transverse prone position across the mouth of the blast tube.	Yes, with chest protection and head exposed	Compressed air	20.63 psi	At days 15 and 44 post blast but not on day 66.	(57)
	SD rats (245–265 g)	Transverse prone position across the mouth of the blast tube.	Yes, with chest protection and head exposed	Compressed air	20.63 psi	Inconclusive	(82)
	Male and naturally cycling female C57BL/6J mice (7–9 weeks of age)	Prone position facing blast exposure	Yes	Compressed air	15.74 Psi	At 6 days post blast in male mice. Female mice had deficits that did not reach significance.	(83)
	LE rats (250 g−350 g; 10 weeks of age)	Head facing the blast exposure	Yes	Compressed air	74.5 kPa	At 30 weeks post blast	(65)
	SD rats (60–70 days)	503 cm from the bursting membrane and 112 cm from the open end of the driven cylinder	Not mentioned	Helium	14 Psi	At 5 weeks post blast.	(53)
Staircase test	ICR Mice (25–30 g)	4 and 7 m away from detonation source	No	500 g TNT	Shockwave and reflected wave 2.5Psi or 5.5 Psi	Up to 30 days for both groups of animals	(12)
Elevated Zero Maze	Adult C57BL/6 mice (12–16 weeks)	1 cm outside exit end of blast tube facing head front.	both head restrained and unrestrained	Helium	Mild blast of 215 ± 13 kPa, 46 ± 5 kPa * ms impulse or moderate blast of 415 ± 41 kPa, 148 ± 12 kPa * ms impulse	No significant deficits in both blast groups vs. sham 24 h post blast.	(66)
	LE rats (250–350 g, 10 weeks old)	30 cm inside the shock tube with head facing the blast exposure	Yes	Compressed air	74.5 kPa for 3 days	At 24 weeks post blast.	(50)
	LE rats (250–350 g, 10–12 weeks old)	Head facing the shockwave exposure	Yes	Compressed air	One 74.5 kPa exposure for 3 consecutive days	At week 11 post blast.	(62)
	LE rats (250–350 g)	18 inches inside the shock tube, both facing and sideways to the blast	Yes	Compressed air	74.5 kPa for 3 days	3 rats had deficits while 9 did not at 3 weeks post injury. 9 unaffected and 4 affected rats at 6-month timepoint.	(84)
Open Field test (OFT)	Adult C57BL/6 mice (12–16 weeks)	1 cm outside exit end of blast tube facing head front.	Both head restrained and unrestrained	Helium	Mild blast of 215 ± 13 kPa, 46 ± 5kPa * ms impulse or moderate blast of 415 ± 41 kPa, 148 ± 12 kPa * ms impulse	At day 4 post blast	(66)
	C57BL/6J mice (2-month-old)	Prone position 3, 4, and 7 m away from the source with mice head longitudinally aligned to the shockwave propagation	No	C4 explosives	~46.6 kPa, ~31.9 kPa and ~19.6 kPa corresponding to 3, 4, and 7 m distances from source.	At 6 days post injury in the 3 m blast group	(58)
	SD rats (215–300 g)	73 cm inside the shock tube in an antero- posterior axis with head perpendicular to the shockwave	Yes	Compressed air	3 blasts of 40 Psi; each blast was administered at an interval of 14 days	At days 1 and 7 post blast	(85)
	SD rats (300 g)	19 cm from the nozzle opening at an angle of 21° from the vertical axis of the shock tube.	Not mentioned	Nitrogen gas	28 kPa	At 3 days post impact	(86)
	SD rats (300–330 g)	Transverse prone position across the mouth of the blast tube.	Yes. Chest protection with rat head exposed	Compressed air	138 kPa single or 5 blasts	Significant deficits on day 1. No deficits were observed on day 16	(19)
	Male and naturally cycling female C57BL/6J mice (7–9 weeks of age)	Prone position facing blast exposure	Yes	Compressed air	15.74 Psi	No deficits	(83)
	Male C57/Bl6 mice (3–4 months; 25.22 ± 1.96 g)	Supine position 53 cm from driven section opening	Yes	Helium	Rupture pressure of 183 kPa(mild) or 213 kPa (moderate)	Moderate blast rats had deficits up to 30 days. Mild injury rats had deficits up to 10 days. Freezing response was observed in mild rats up to 30 days.	(16)
	SD rats (325 g)	Prone position with the right side of the thorax facing the shock wave	Not mentioned	Helium	28.49 ± 1.5 Psi in 2.475 ± 0.16 ms	Significant deficits 6-days post blast	(87)
Forced Swim test	Male SD rats (150–200 g)	265 mm from the exploding wire with head facing impact	No	Exploding wire technique to generate small scale blast waves	95 kPa for 0.189 ms	No deficits were observed	(47)
	Male SD rats (290–320 g, 10–12 weeks)	1 m from the charge in a transverse prone position	Yes	Swedish army plastic explosive containing explosive m/46, 86% pentaerythritol tetranitrate and mineral oil	550 kPa in 0.2 ms	Decreased immobility in blast animals that may be due to Hyperarousal 24 h post blast. No deficits at 14- or 35-days post blast	(88)
	SD rats (6-month-old)	Placed outside with right side of the skull facing the pressure wave	No	Nitrogen	50 Psi in 1 ms	At 72 h post blast	(54)
Light/dark box emergence test	LE rats (250–350 g, 10–12 weeks old)	Head facing the shockwave exposure	Yes	Compressed air	One 74.5 kPa exposure for 3 consecutive days	At week 11 post blast	(62)
	SD rats (300g)	19 cm from the nozzle opening at an angle of 21° from the vertical axis of the shock tube.	Not mentioned	Nitrogen gas	28 kPa	Up to 9 days	(86)
	LE rats (250 g−350 g; 10 weeks of age)	Head facing the blast exposure	Yes	Compressed air	74.5 kPa	At 29 weeks post blast	(65)
	Male SD rats 250-300g	Rostral cephalic orientation toward shock wave	Not mentioned	Compressed helium	10,14, 24 psi	At 7 days post blast in the 10 & 14 psi group.	(89)
	SD rats (250 g)	1.09 m from open end of the blast tube in a rostral cephalic position	Yes. No information on head restraint	Helium	117 kPa at 2.5 ms	Significantly decreased time 1- and 3-months post blast to enter dark box but no difference in time spent or number of transitions	(36)
	C57BL/6J mice (2–month-old)	Prone position 3, 4, and 7 m distances away from the source with the head longitudinally aligned to the shockwave propagation	No	C4 explosives	~46.6 kPa, ~31.9 kPa and ~19.6 kPa corresponding to 3, 4, and 7 m distances from source.	5 days post injury in the 3 m blast group	(58)
	LE rats (250–350 g)	18 inches inside the shock tube, both facing and sideways to the blast	Yes	Compressed air	74.5 kPa for 3 days	Two animals developed deficits while 6 animals showed no deficits, 3 weeks post injury. At 6 months post injury, 9 animals were unaffected while 3 animals showed deficits.	(84)
Acoustic Startle	Male SD rats (150–200 g)	265 mm from the exploding wire with head facing impact	No head restrained; movement restricted	Exploding wire technique to generate small scale blast waves	95 kPa for 0.189 ms	At days 38 and 62 post impact.	(47)
Predator Scent Challenge (open field and light /dark emergence test)	LE rats (250–350 g, 10 weeks old)	30 cm inside the shock tube with head facing the blast exposure	Yes	Compressed air	74.5 kPa for 3 days	At 3 days in open field test and at 7 days in light/dark emergence task post exposure to predator scent	(50)

The open field test (OFT) assesses the animal's locomotor activity, exploratory behavior and anxiety. Briefly, the test involves placing the animal in a square, rectangle or circular box with set spacing requirements as described by Gould et al. (91), Seibenhener et al. (92), and recording the animal's exploratory behavior (19, 46, 91, 92). Two factors are known to influence anxiety-like behavior in the open field. The first is the social isolation resulting from the physical separation from cage mates when performing the test. The second is the stress/aversion created by the brightly lit, unprotected, novel test environment (92). Rodents exposed to the novel experimental arena typically spend greater time exploring the periphery rather than the center area. This tendency of animals in known as thigmotaxis. Mice or rats exploring more time at periphery of the arena than the center represents anxious behavior. Besides exploratory activity other anxiety behaviors such as grooming, rearing and defecation can be recorded in OFT task.

The light/dark box test consists of a dark box which represents the “safe area,” a light box which represents the aversive environment and utilizes the rodent's innate aversion to light and exploratory behavior to measure anxiety. The animal is habituated in a dark room and placed in the center of the light box and recorded. The time taken by the animal to explore and reach the dark box is measured. Animals with anxiety are generally observed to take more time to reach the dark box than control animals. This test has been identified to be more effective when done in the dark or night cycle of rodents as their plasma corticosterone levels which are higher in their dark cycle play a significant role in their exploratory behavior (93).

One of the major pitfalls involved in anxiety testing is habituation. Testing done over multiple timepoints leads to habituation of the new/aversive environment leading to a decline in the exploratory behavior of the rats. The weight of the animals and motor deficits have also been identified to play a negative role in their exploratory behavior (37, 73, 90).

Table 2 summarizes the work done in literature regarding anxiety and depression behavior testing in rodents subjected to bTBI. Literature review was done using advanced search in Google Scholar with keywords “rodents, shockwave tube, anxiety, depression” and the exact phrase “blast TBI,” between the years 2008-2019. In most published works in Table 2, bTBI has been correlated with positive anxiety deficits in rodents.

Anxiety measured by the elevated plus maze, light/dark emergence test were identified in most studies at chronic timepoints of 7, 15 days post bTBI resulting in long term deficits irrespective of animal location in the blast tube (36, 48, 56, 65). However, no deficits were observed in animals placed 7 m away from detonation source in open field explosions (59, 60). Animals exposed to multiple blasts analyzed with the elevated zero maze were identified with deficits that lasted months post exposure. Deficits in the open field test were observed 1–7 days post injury in animals placed outside the blast tube (66, 85). Chronic deficits lasting a month have not been reported with the open field test except in a study involving moderate bTBI overpressure (16). These results implicate the importance of experimental parameters and choice of behavior tests in measuring deficits following bTBI.

### Fear Conditioning

PTSD and bTBI have symptoms that often overlap with each other. These include attention deficits, irritability, increased startle response, sleep disturbance, emotional numbness and anger (94, 95). However, there is also increasing evidence indicating the development of chronic PTSD as a result of secondary neuronal damage following a bTBI (96–98). PTSD is known to manifest in the following stages: an individual is first exposed or witness to a life-threatening situation leading to re-experiencing symptoms such as nightmares and sense of reliving the trauma. This then leads to active avoidance of the trauma, inability to recall the trauma, emotional numbing resulting in irritability, insomnia, hyper vigilance, or an increased startle response (95). PTSD is associated with medial and orbitofrontal cortices, amygdala, and hippocampal regions of the brain (97). Fear conditioning is one of the tests that is used to measure PTSD traits in rodent models of bTBI. Contextual and cued fear conditioning test involve the amygdala, hippocampus, frontal, and cingulate cortex brain regions (99).

Contextual and cued fear conditioning is done by training the rodent in a chamber (context) and providing a conditional stimulus (CS) typically an aversive noise followed by an unconditioned stimulus (US) such as a foot shock. The pairing of the CS- US generates the freezing behavior in rats. When this pairing is repeated, the animal creates a stronger negative association to the testing environment and the auditory cue. This then generates a freezing response when the animal is returned either to the testing chamber or on hearing the auditory cue. The contextual fear memory is tested by placing the rat in the same chamber and measuring the freezing behavior in the animal without the presence of the CS or US. In order to test the cued conditioning, the animal is placed in a different chamber and its freezing response to the CS is now measured. This enables to differentiate the freezing effect observed due to the context and the CS (50, 99).

Table 3 summarizes the fear conditioning tests done in bTBI literature using Google scholar and keywords “shockwave tube,” “rodents,” “fear conditioning,” and the exact phrase “blast TBI” between the years 2008–2019. Most of the published studies in bTBI measuring fear conditioning post bTBI were carried out with repetitive low-level blasts and have consistently reported an increase in freezing or cued fear response following bTBI. The duration of deficits have been reported from 1 to 25 weeks post blast. Fear conditioning deficits measured in a single blast exposure by Perez Gracia et al. (65), were observed 3 weeks post injury further implicating the long-term consequence of blast exposure on fear conditioning (65). These results indicate the development of PTSD related deficits following bTBI in rodent models.

**Table 3:** Fear conditioning behavioral testing in bTBI.

**Behavior test**	**Rodent used**	**Position of animal**	**Head restraint**	**Gas used in blast tube**	**Blast Pressure**	**Duration of deficits**	**References**
Fear conditioning	SD Rats 325 g	Head facing blast impact placed inside the blast tube	Yes	Compressed air	Three 74.5 kPa, 4.8 ms exposures, one each day	Significant reduction conditioned fear suppression one-week post impact	(100)
Cued and contextual fear conditioning	LE rats (250–350 g, 10 weeks old)	30 cm inside the shock tube with head facing the blast exposure	Yes	Compressed air	74.5 kPa for 3 days	Significant freezing in cued testing 25 weeks post impact	(50)
	LE rats (250–350 g, 10–12 weeks old)	Head facing the shockwave exposure	Yes	Compressed air	One 74.5 kPa exposure for 3 consecutive days	Between weeks 11–17 post blast	(62)
	LE rats (250 g–350 g; 10 weeks of age)	Head facing the blast exposure	Yes	Compressed air	74.5kPa	Cued fear response was significantly enhanced at 35 weeks post blast.	(65)
	LE rats (250–350 g)	18 inches inside the shock tube, both facing and sideways to the blast	Yes	Compressed air	74.5 kPa for 3 days	8 rats did not develop deficits, 4 rats developed deficits 3 weeks post injury.	(84)
Grimace scale	Male and naturally cycling female C57BL/6J mice (7– 9 weeks of age)	Prone position facing blast exposure	Yes	Compressed air	15.74 Psi	No deficits 6 days post blast	(83)

### Motor Deficits

Veterans diagnosed with bTBI have been known to manifest balance and vestibular motor co-ordination issues with symptoms ranging from dizziness, vertigo, postural instability and impaired tandem gait (101–103). Further, balance deficits are found to be amplified in veterans with a combined diagnosis of TBI and amnesia or PTSD (104). Balance and gait disorders in rodent bTBI models are measured using staircase test, ladder test, rotarod, and beam balance test as described in Table 4. Additionally, anxiety assessment apparatus such as elevated plus maze and open field test have also been used to measure locomotor deficits in rodents.

**Table 4:** Summary of experimental variables and motor deficit outcomes in bTBI.

**Behavior test**	**Rodent used**	**Position of animal**	**Head restraint**	**Gas used in blast tube**	**Blast pressure**	**Duration of deficits**	**References**
Staircase test	ICR Mice (25–30 g)	4 and 7 m away from detonation source	No (animals were fixed in position with plastic net)	500 g TNT	Shockwave and reflected wave 2.5 Psi or 5.5 Psi	Up to 7 days only in the 2.5 Psi group. No deficits were observed at 30 days for both groups	(12)
Ladder test	Male SD rats (250g)	1.09 m inside the shock tube facing impact	Yes. No information on head restraint	Compressed helium	97, 117, 153 kPa	No deficits were observed at 3, 6, 48 or 72h post blast	(17)
Beam walk task	SD rats (250–350 g)	Transverse prone position across the mouth of the blast tube.	Yes	Compressed air	114, 126, and 147 kPa	Up to 3 days	(30)
	SD rats (200–250 g)	Prone position 20cm from the end of the tube facing the blast wave	No	Compressed air	74 kPa	No deficits up to 14 days post blast.	(63)
Beam walk/ Beam balance test	Adult male SD (350-480g)	Transverse prone position with the head perpendicular to the direction of the shockwave.	Yes	Compressed air	135 ±12.4 kPa with a mean duration of 3.50 ±0.063 ms	No significant deficits 1–5 days post blast.	(105)
Photo beam walk	Male SD 350 g rats	Outside the blast tube with the right side of head perpendicular to the blast front.	No	Compressed nitrogen gas.	Reflected pressures of 31.47, 50.72, and 72.05 in <3 ms	At 24 h for all blast groups	(106)
Beam balance test	LE rats (250–−300 g)	18 inches inside the shock tube, both facing and sideways to the blast	Yes	Compressed air	36.6, 74.5, and 116.7 kPa	No deficits in the 36.6 and 74.5 kPa rats in both orientations. The rats in frontal orientation at 116.7 kPa showed significant deficits at 30 min which resolved by 2 h. Side-oriented rats had deficits at 30 min and 2 h that was resolved by 6 h	(18)
	Male Wistar rats (408.3 ± 93g)	Right side of the front cortex is aligned to the impact	Yes	Lithotripsy machine fired 5 shockwave pulses with 60 Hz frequency	n/a	Up to 7 days post impact	(81)
	Heterozygous *WldS* mice (8-week-old)	10 cm from Mylar membrane with the left side of head facing the blast	No	Compressed air	27kPa	Day 28 post blast	(55)
Rotarod test	Male C57/Bl6 mice (3–4 months; 25.22 ± 1.96 g)	Supine position, 53 cm from driven section opening	Yes	Helium	Rupture pressure of 183kPa(mild) or 213 kPa (moderate)	Up to day 14 but not on days 21 and 30 in both groups	(16)
	Male C57BL/6J mice (8–10 weeks old; 22–26 g)	2.5 feet inside the open end of the expansion chamber, perpendicular to the direction of the shock wave	Yes	Compressed air	Two 20.6 Psi blasts separated by a min and a third blast 30 min later	Significant deficits in single and repetitive blast animals at 10 rpm, 2 and 24 h post blast.At 20 rpm, single blast animals had deficits up to 72 h and multiple impact animals up to 120 h.	(107)
	Adult C57BL/6 mice (12–16 weeks)	1 cm outside exit end of blast tube facing head front.	Experiments were conducted with both head restrained and unrestrained	Helium	Mild blast of 215 ± 13 kPa, 46 ± 5kPa * ms impulse or moderate blast of 415 ± 41 kPa, 148 ± 12 kPa * ms impulse	No deficits in both groups of blast animals up to 3 days post blast	(66)
	LE rats (250–350 g, 10 weeks old)	30 cm inside the shock tube with head facing the blast exposure	Yes	Compressed air	74.5 kPa for 3 days	No motor deficits were observed in rotarod at 8 weeks post blast.	(50)
	SD rats (300 g)	At 19 cm from the nozzle opening at an angle of 21° from the vertical axis of the shock tube.	Not mentioned	Nitrogen gas	28 kPa	Up to 9 days post blast	(86)
	SD rats (200–250 g)	Prone position 20 cm from the end of the tube facing the blast wave	No	Compressed air	74 kPa	No deficits up to 14 days post blast.	(63)
	Male Wistar rats (408.3 ± 93g)	Right side of the front cortex is aligned to the impact	Yes	A lithotripsy machine fired 5 shockwave pulses with 6o Hz frequency	n/a	Up to 7 days post impact	(81)
Open Field Test (OFT)	SD rats	39 and 17 cm outside shock tube opening, 40 and 20° lateral to shock tube axis, respectively	yes	Helium	100 and 450 kPa reflected peak overpressure	At 4 days and 30 days post impact in both blast groups	(46)
	SD rats (~300 g)	Transverse prone position across the mouth of the blast tube.	Yes, with chest protection and head exposed	Compressed air	20.6 ± 3 psi	Higher resting times at day 45 post injury.	(56)
	Adult C57BL/6 mice (12–16 weeks)	1 cm outside exit end of blast tube facing head front.	Experiments were conducted with both head restrained and unrestrained	Helium	Mild blast of 215 ± 13 kPa, 46 ± 5 kPa * ms impulse or moderate blast of 415 ± 41 kPa, 148 ± 12 kPa * ms impulse	At day 4 post blast in both groups.	(66)
	C57BL/6J mice (2-month-old)	Prone position 3, 4, and 7 m distances away from the source with the head longitudinally aligned to the shockwave propagation	No	C4 explosives	~46.6 kPa, ~31.9 kPa and ~19.6 kPa corresponding to 3, 4, and 7 m distances from source.	At 3 days post injury in the 3, 4, and 7 m blast group	(58)
	LE rats (250–350 g, 10 weeks old)	30 cm inside the shock tube with head facing the blast exposure	Yes	Compressed air	74.5 kPa for 3 days	No motor deficits at 7 weeks post blast.	(50)
	LE rats (250–350 g, 10–12 weeks old)	Head facing the shockwave exposure	Yes	Compressed air	One 74.5 kPa exposure for 3 consecutive days	No deficits 11 weeks post blast	(62)
	SD rats (300–330 g)	Transverse prone position across the mouth of the blast tube.	Yes. Chest protection with rat head exposed	Compressed air	138 kPa single or 5 blasts	At day 1 but not on day 16 in multiple impact group.	(19)
	C57BL/6 Mice (2.5 months)	0.56 m inside open exit of shock tube	Both with and without head restraint	Compressed gas	77 ± 2 kPag	No deficits	(26)
	LE rats (250 g−350 g; 10 weeks of age)	Head facing the blast exposure	Yes	Compressed air	74.5kPa	At 28 weeks post blast	(65)
Elevated plus maze	SD rats (~300 g)	Transverse prone position across the mouth of the blast tube.	Yes with chest protection and head exposed	Compressed air	20.63 psi	At day 44 post blast but not at days 15 or 66.	(57)
	SD rats (245–265 g)	Transverse prone position across the mouth of the blast tube.	Yes, with chest protection and head exposed	Compressed air	20.63 psi	Immediately after blast.	(82)

The Rotarod test originally described by Dunham and Miya is often used for testing the neurological effects of drugs or trauma on the motor coordination of rodents (50, 108–110). The apparatus consists of a rotating rod of constant or accelerating speed on which the rodent is placed and variables such as the time and the speed of rotation for the animal to fall from the apparatus is noted and evaluated for motor deficits. The Rotarod apparatus was identified to be more sensitive in identifying motor deficits in mild TBI than the beam balance or beam walk test (108). The disadvantages of this model are that the deficits observed in the rotarod test can also be influenced by endurance and motor learning in the animals. Motor learning refers to the rodent's ability to develop a strategy to remain on the rotating rod rather than as a result of improved locomotor skills (111, 112). Stress has also been identified to play a role in rotarod deficits (113).

The beam walk, ladder rung tests measure the fine motor skills of the rat unlike the gross motor skills assessed by the rotarod test (99). The beam walk or beam balance apparatus consists of a narrow-elevated beam that the rodent is made to walk on from one end to the other. The number of foot faults of the rodent is measured to assess its motor performance. The advantages of the test are the low cost, the relative ease of setup and very little motor learning associated with recovery of function. However, the weight of the animal can affect their performance on this apparatus. Beam walking test is more suited to identify deficits in moderate and severe TBI than in mild TBI (112). The ladder rung test, similar to the beam balance test, measures the foot faults of rodents as they walk across a horizontal ladder to reach a platform on the other side. The ladder rung test, additionally, allows to assess the grasping ability and motor deficits in each limb of the rodent. The difficulty of the test can be varied by altering the distance between the rungs. An irregular pattern of rung placement for each trial prevents familiarity and learning in the rodents (114). One disadvantage of the beam walk and ladder rung tests is the manual assessment of foot fault scoring which can be tedious and may differ from one individual to another. Anxiety tests such as open field test has also been utilized to study locomotor deficits. Ambulation, latency and rearing of the rodents can be assessed through these tests in addition to anxiety and depression traits (92).

Table 4 summarizes literature review done using Google scholar and keywords “shockwave,” “rodents,” “motor deficits,” and the exact phrase “blast TBI” between the years 2008–2019. Motor deficits, similar to the spatial working memory deficits, have not been consistently reported in bTBI. Most of the deficits observed following bTBI were in animals placed at the exit end of the blast tube implicating the role of reflective pressures and tertiary loading of the blast (30, 46, 86). Majority of the studies with animals located inside the blast tube have reported no motor deficits (17, 18, 50). However, animals located inside the blast tube subjected to multiple blasts have been identified to develop motor deficits (107).

### Auditory Deficits

Blast TBI has been well-associated with tinnitus and hearing loss deficits in the veteran population (115–117). In a study done by Oleksiak et al. (118), with a sample population of veterans with mild TBI, 76% of veterans were associated with tinnitus while 60% developed hearing loss. About 92.5% of personnel exposed to blast TBI reported some form of hearing loss (118). Blast exposure can cause both peripheral and central auditory system damage by rupture of the tympanic membrane, ossicular disruption, structural damage of inner and outer hair cells and impairment of central auditory processing (CAP). Blast related auditory deficits include CAP deficits, peripheral hearing loss, tinnitus, and vestibular deficits. Tinnitus is defined as head or ear noise that lasts 5 min or longer while CAP deficits causes difficulty in hearing background noises, speech perception, sound localization, and lateralization (4).

Auditory deficits in bTBI rodent models have been evaluated using acoustic startle response (ASR) and various modifications of the test to include prepulse inhibition for identifying hearing loss and gap detection deficits for evaluating tinnitus. The acoustic startle response is described as a rapid contraction of facial and skeletal muscles in response to a loud and unexpected auditory stimulus usually 80 dB above the auditory threshold for a rat (119). The acoustic signaling pathway consists of the auditory nerve, the ventral cochlear nucleus, the cochlear root neurons, the caudal pontine reticular nucleus (PnC), spinal interneurons and finally the motor neurons which produce the startle response (119–121). The PnC neurons, specifically, play a crucial role in the startle response elicited (122, 123).

The acoustic startle experiments are generally conducted in a chamber mounted with a platform containing an accelerometer or other voltage motion sensors to assess the startle response in rats. Briefly, the test involves acclimatizing rats to background noise (65–68 dB) before introducing the animal to increasing intensity of the startle stimuli. The startle amplitude of rats is then measured as an output function of the startle intensity provided. For the prepulse inhibition, a sound of lower intensity than the startle stimulus is presented before the startle stimuli. This prepulse stimulus provided in anticipation of the startle stimulus is expected to lower the startle amplitude in control subjects but not in subjects with hearing loss (124). A modification of this paradigm is the gap prepulse inhibition which has recently gained prominence in evaluating tinnitus in human and animal models. The gap prepulse inhibition involves a gap trial in which a silent gap is provided prior to the startle stimuli. Since subjects with tinnitus are expected to fail to hear the silent gap, there would be no reduction in startle amplitude in the gap trials when compared to control subjects. The gap detection is measured as the ratio between the magnitude of the startle stimulus presented alone without a gap trial and trials in which a gap preceded the startle stimulus (125).

The main advantage of the acoustic startle test is since it measures the reflex of the rodent, there is no training involved prior to conducting the test. However, there is considerable variability in the experimental protocol and data interpretation for the gap detection experiments across laboratories. One of the major discrepancies is in comparing the “no gap” vs. “gap trials” of individual rats and determining the presence of tinnitus than comparing the overall performance across groups. Since it is possible that only a percentage of blast exposed rats may develop tinnitus, it would be beneficial to compare individual rat performances than performances across groups (125). Further, there has been contradicting evidence in the assumption that subjects with tinnitus would “fill in” the silent gap trial (126, 127). However, there is significant evidence to support that the gap detection ratio measured is affected in rats with tinnitus (127, 128).

Blast induced tinnitus and hearing loss investigations done in rodent models are summarized in Table 5 using Google Scholar and keywords such as “blast TBI,” “rodents,” “hearing loss,” “tinnitus,” and the exact phrase “blast TBI” between the years 2008–2019. Most of the studies at different blast overpressures observe consistent findings of hearing loss and tinnitus in blast exposed animals indicating the prevalence of auditory deficits in bTBI and the efficacy of the behavioral test.

**Table 5:** Auditory deficits in bTBI.

**Behavior test**	**Rodent used**	**Position of animal**	**Head restraint**	**Gas used in blast tube**	**Blast pressure**	**Duration of deficits**	**References**
Prepulse inhibition (PPI) and acoustic startle	LE rats (250–350 g, 10–12 weeks old)	Head facing the shockwave exposure	Yes	Compressed air	One 74.5 kPa exposure for 3 consecutive days	Significant increase in PPI in blast animals between weeks 11–17 post blast	(62)
	SD Rats (225–250 g)	Supine position with the blast wave generator nozzle right above the animal's head.	Yes, chest shielded with 2 mm lead plate	Nitrogen	80 Psi	Decreased startle amplitude response 2 weeks post blast but no effects of PPI	(31)
	LE rats (250–350 g, 10 weeks old)	30 cm inside the shock tube with head facing the blast exposure	Yes	Compressed air	74.5 kPa for 3 days	Increased acoustic startle 24 weeks post blast but had no change in PPI	(50)
	LE rats (250 g−350 g; 10 weeks of age)	Head facing the blast exposure	Yes	Compressed air	74.5 kPa	Enhanced PPI at 34-week post blast	(65)
Gap detection (GAP), acoustic startle reflex and PPI	SD Rats (70–80 days)	1.09 m from open end of blast tube in a rostral cephalic orientation	Yes, with one ear occluded. No information on head restraint	Helium	150 kPa for 10ms	Tinnitus positive rats had impaired GAP ratios 24 h, a month and 3 months post blast. PPI ratios were increased only at the 24 h time point. All blast rats had decreased startle force at 24 h, 1 month, and 3-month, time points.	(129)
	SD rats (110 days old, 250-300g)	109 cm inside the open end of the blast tube in a rostro-cephalic orientation to the blast wave	Not mentioned. Right ear was occluded with an earplug.	Helium	3 blast exposures of 22 psi 5 min apart.	Hearing loss and tinnitus up to 7 weeks post blast	(128)
GAP and PPI testing.	Long Evans rats (60–70 days old)	44 inches from open end of tube in a prone position with head facing blast	No earplugs. Head restraint not mentioned	Helium	95 kPa for 10 ms	Tinnitus observed at 24 h and at certain frequencies on day 14. The GAP deficits recovered by day 28. PPI deficits pertained at particular frequencies up to day 90.	(130)
	SD rats (60–70 days)	503 cm from the bursting membrane and 112 cm from the open end of the driven cylinder	Yes, with no earplugs. Head restraint not mentioned	Helium	14 Psi	8 out of 13 rats developed tinnitus,	(53)

*Behavioral tests to measure hearing loss and tinnitus in bTBI rodent models*.

## Summary and Discussion

The primary effects of blast TBI have been replicated and studied in rodent models by many researchers. We have attempted in this review to summarize the various behavioral models used to study common behavioral outcomes of blast TBI such as cognition, anxiety, depression, PTSD, motor, and auditory deficits. The advantages and shortcomings of the tests most commonly used in literature have been summarized in Table 6.

**Table 6:** Behavior tests used in blast TBI with their advantages and disadvantages.

**Behavioral tests**	**Advantages**	**Disadvantages**
**Cognition:**		
• Morris Water Maze • Barnes Maze • Y Maze • Novel Object Recognition • Active avoidance test	Fear of water serves as a good motivator, minimal training required, most rodents learn to perform the test. Does not induce stress in animals Simplicity and lack of training Simple setup, no training required and less stressful Less time required for testing	Induces stress in animals. Depressed animals can exhibit “floating” behavior that can affect results Lack of motivation may result in poor performance. Stress can affect results observed Inconsistent exploration of the novel object amongst animals of the same group. Rodent results within same group can be inconsistent.
**Anxiety and depression:**		
• Elevated Plus Maze • Elevated Zero Maze • Open Field Test • Light/dark box emergence test • Forced Swim Test	Simplicity of testing, no training required. Simplicity of testing, no training required. Quick and easy to use, no prior training required. Ease of testing and analysis.	Testing at multiple timepoints results in habituation and false positive results. Locomotor deficits can also affect results. Locomotor deficits affects results. Weight of animals can affect their exploratory behavior. Locomotor deficits can affect results. The lit side of the test has to be sufficiently aversive to the rodents to avoid false positive results. Locomotor deficits have to be considered to prevent false positive results.
**Fear conditioning:**		
• Cued and contextual fear conditioning	Motor deficits do not affect the results as the test involves passive learning.	The strain and phenotype of rodents have to be taken into consideration for the cue and aversive conditioning levels.
**Motor deficits:**		
• Beam Walk/Beam Balance Test • Rotarod	Low cost, relative ease of setup, very little motor learning associated with recovery function. Highly reproducible results	Weight of the animal may affect results. Manual assessment of foot fault scoring can be tedious and produce inconsistent results. Factors such as age, weight of the animals, lack of motivation affects results. Recovery of function observed can be influenced by endurance and motor learning in animals. Training of animals is required to reduce stress and to segregate rodents capable of performing on the rotarod.
**Auditory deficits:**		
• Acoustic startle	No prior training as it measures the reflex of the animal.	Variability in data interpretation across labs.

Cognitive deficits in blast induced TBI have been studied using spatial memory tasks such as Morris Water maze, Y maze, Barnes Maze, and object recognition tasks such as the novel object recognition test. From Table 1, it can be inferred that animals exposed to the reflective blast pressure or blast wind manifest spatial memory deficits. In the studies where the animals were placed inside the blast tube, a few researchers observed deficits with Morris Water Maze test at 24 h post impact but not at longer timepoints. No cognitive deficit was observed with the eight arm radial maze. Multiple blast studies done with the Morris Water Maze test also indicated similar transient deficits at 24 h but not at later timepoints (49, 50). Studies done with animals placed outside the blast tube, on the other hand, consistently reported cognitive deficits lasting for weeks indicating these animals may be receiving a higher severity of TBI (30, 31, 57). Investigations of bTBI with animals placed outside the shock tube have demonstrated that the animals are not only subject to the pure effects of a primary blast wave but can be subject to the reflective pressures and blast wind (10, 22). Similarly, spatial memory deficits were also observed to last for weeks or months in animals subject to open field detonations (12, 58). Stemper et al. (48), reported no cognitive deficits up to 60 days post impact when animals were placed at an angle to the opening of the blast tube to minimize the blast wind effects (48). These results indicate that chronic spatial memory deficits observed due to a blast impact are not majorly manifested due to the primary blast wave and could be a result of the secondary or tertiary effects of a blast impact. This was further demonstrated by Goldstein et al. (26), when spatial memory deficits were observed in animals subject to bTBI without head restraint but not in animals with head restraint (26). Thus, it is possible that the spatial memory deficits are more likely to be produced due to tertiary effect of head and body motion and not primary loading effect when the animal is relatively motionless. However, this hypothesis has not been carefully tested.

Object recognition memory, however, measured by the novel object recognition test showed consistent deficits in animals placed both inside and outside the blast pressure tube indicating that primary blast pressure does alter the recognition memory in rodents. Interestingly, spatial object recognition deficits were not affected by the presence or absence of head restraint unlike the spatial working memory deficits (66). This further supports that primary blast loading affects recognition memory while the effects of tertiary loading may affect the working memory. Fear based learning tests such as the active avoidance test which is associated with deficits in the basal forebrain also showed deficiencies following the primary blast injury (18, 29, 131).

Anxiety due to bTBI has most commonly been studied using elevated plus maze, zero maze, open field test and light/dark box emergence tests. In Table 2, most of the anxiety deficits measured by these tests are seen to be manifested at chronic timepoints with the quickest onset being 4 days post blast in the OFT test and not as immediate deficits post blast exposure. Most of the studies using EPM were done with the animals placed at the mouth of the blast tube manifesting chronic anxiety deficits. However, studies by Perez-Garcia et al. (120), and Russell et al. (83), have also demonstrated chronic anxiety deficits in animals subjected to bTBI placed inside the shock tube (65, 83). Statz et al. (84), subjected rats to a repetitive mild blast TBI of 74.5 kPa over 3 consecutive days and found that a part of the blast cohort, but not all animals, manifested anxiety measured by elevated zero maze, light/dark emergence test and fear conditioning, 3 weeks and 6 months post blast impact (84). Increased anxiety behavior at 24 hrs to 3 days post blast exposure was observed in the open field test only when the animals were placed at the exit end of the blast tube or were subjected to multiple blast exposures over days (19, 54, 66, 85, 86). EPM and light /dark emergence tests showed long term deficits of anxiety following bTBI and may be more accurate tests for anxiety. Open field tests are also dependent on exploratory behavior of rodents but deficits in this model were observed only at early timepoints of few days post blast and no deficits were observed at chronic timepoints of a month post blast.

Increased fear conditioning has been observed in blast animals at chronic time points from single blast or multiple blast exposure (Table 3). Interestingly, a decrease in fear conditioning was observed in repetitive bTBI in a study by Genovese et al. (100). Auditory deficits that could play a role suppressing the ability to hear the aversive cue and thereby the performance of the animals in the fear conditioning test were negated as there were no auditory deficits observed in the prepulse inhibition test. However, in this study conditioned suppression was measured and not the fear conditioning. Conditioned suppression is the reduction of the conditioned response to a positive stimulus, which in this study was a lever press to obtain food when the aversive stimulus was presented. Hence, the conditioned suppression parameter measured in this study might produce different results than the typical fear conditioning measured in other studies (100). Whether conditioned fear and conditioned suppression are correlated or different remains disputable and hence the results may still indicate deficits to the conditioned fear in the blast animals (100, 132–134).

Motor deficits following bTBI were measured by Rotarod, beam walk/balance tests, elevated plus maze and open field tests. Motor deficits in bTBI showed a similar trend to the cognitive deficits, with increased deficits in animals exposed to the reflective pressures, placed at the mouth of the blast tube or placed inside an open-ended shockwave tube. Most of the studies with animals placed inside the tube indicated little to no motor deficits.

Auditory deficits in blast TBI have been measured using the acoustic startle test with prepulse inhibition for identifying hearing loss and gap detection deficits for evaluating tinnitus. bTBI induced hearing loss and tinnitus have consistently been reported as chronic deficits. However, some studies report only a part of the cohort undergoing blast to be associated with hearing deficits and tinnitus (Table 5). It is critical to make this distinction between animals with and without auditory deficits within the injury group to avoid possible dilution of results.

Overall, it is observed from Tables 1–5, that the blast pressures used in most studies investigating behavioral deficits of bTBI are mild blasts ranging from 70 to 153 kPa. The most consistently reported chronic behavioral deficits following primary blast TBI in literature between the years 2008–2019 were anxiety, auditory deficits, recognition memory deficits, and fear conditioning often observed between a week to 1-month post blast exposure. This indicates that exposure to primary mild bTBI can lead to persistent anxiety, auditory, fear conditioning and recognition memory deficits. Additionally, animal location in the blast tube did not play a significant role in the development of these deficits. Spatial working memory and motor deficits following bTBI, on the other hand, depend heavily on experimental parameters. Exposure to a primary blast wave where the animal is kept inside the shock tube seemed to cause only transient effects on working memory measured by the Morris Water maze lasting 24 h post injury while exposure to tertiary effects of blast, reflective pressure wave leads to long lasting deficits. Similarly, motor deficits in a primary blast exposure causes little to no deficits while manifesting long term deficiencies in open field experiments and in studies where animals are placed at the exit end of the blast tube. This suggests that the primary blast wave exposure has a significant role in the manifestation of recognition memory deficits, auditory, fear conditioning and anxiety deficits while tertiary effects of loading experienced by the animal placed outside the blast tube may have a role in the manifestation of spatial working memory and motor deficits. Thus, position of the animal in the blast tube and use of head restraint plays a significant role in bTBI behavioral deficits and have to be considered while studying the effects of a primary blast wave.

## Author Contributions

AA carried out the literature research, acquisition of data for the tables, and prepared the manuscript. AR aided in data acquisition for the tables and wrote the subheading Anxiety and Depression in the manuscript. NC and BP procured funding, supervised, provided critical revisions, and edited the manuscript. All authors contributed to the article and approved the submitted version.

## Conflict of Interest

The authors declare that the research was conducted in the absence of any commercial or financial relationships that could be construed as a potential conflict of interest.
